# Reduced EZH2 Expression in Circulating CD8‐Positive T Cells and Monocytes in Psoriasis

**DOI:** 10.1111/exd.70207

**Published:** 2026-01-10

**Authors:** Toyoki Yamamoto, Rino Toyoshima, Lixin Li, Asumi Koyama, Shinichi Sato, Sayaka Shibata

**Affiliations:** ^1^ Department of Dermatology, Graduate School of Medicine The University of Tokyo Tokyo Japan

**Keywords:** EZH2, monocytes, psoriasis, T cells

## Abstract

Enhancer of Zeste Homologue 2 (EZH2) is an epigenetic regulator involved in immune cell differentiation and function; however, its role in psoriasis remains unknown. This study aimed to evaluate EZH2 expression in peripheral blood mononuclear cells from patients with psoriasis and explore its potential functional relevance to disease pathogenesis. Peripheral blood samples were obtained from 40 psoriasis patients and 18 healthy controls, and EZH2 expression in T cell and monocyte subsets was analysed by flow cytometry. EZH2 expression was significantly reduced in circulating CD8+ naïve and memory T cells, as well as in monocyte subsets from psoriasis patients compared to healthy controls. EZH2 levels in CD8+ naïve T cells showed a significant inverse correlation with disease severity scores. Functional analyses revealed that pharmacological EZH2 inhibition suppressed IL‐17A expression in peripheral blood mononuclear cells under IL‐23/IL‐1β stimulation. In addition, immunofluorescence staining identified EZH2‐positive T cells and monocytes within psoriatic skin lesions. Collectively, these findings suggest that EZH2 may be involved in the regulation of type 3 inflammatory responses and may therefore represent an epigenetic regulator contributing to psoriasis pathogenesis.

## 1 Introduction

Psoriasis is a disease driven by immune dysregulation, with T cells and monocyte‐derived cells, such as dendritic cells and macrophages, playing central roles in its pathogenesis. Among helper T (Th) cells, Th17 cells have long been considered the main contributors to psoriasis pathogenesis, primarily through their release of pro‐inflammatory cytokines such as IL‐17 [1, 2, 3] However, accumulating evidence has highlighted the pivotal roles of CD8+ T cells in disease pathogenesis [4, 5, 6]. CD8+ T cells produce IL‐17, and their infiltration into the skin correlates with disease severity. Monocytes, in parallel, amplify skin inflammation by producing cytokines such as TNF‐α, IL‐1β and IL‐6 [7]. In the presence of IFN‐α, monocytes can differentiate into dendritic cells (DCs) or macrophages, which produce IL‐23, a cytokine essential for Th17 cell activation [8, 9, 10]. Additionally, monocytes express CCR2, allowing them to migrate to inflamed skin, where they differentiate into macrophages and contribute to lesion formation [11, 12]. Collectively, T cells and monocytes and their differentiated cell types establish the inflammatory environment characteristic of psoriasis and drive disease progression. The high clinical efficacy of IL‐17 and IL‐23‐targeted antibody therapies underscores the importance of these immune cells in psoriasis pathogenesis [13, 14, 15, 16, 17, 18, 19].

Enhancer of Zeste Homologue 2 (EZH2), the catalytic subunit of Polycomb Repressive Complex 2 (PRC2), is a key epigenetic regulator that mediates histone H3 lysine 27 trimethylation (H3K27me3) to control gene expression [20, 21]. EZH2 has been shown to play a pivotal role in T cell differentiation and polarisation by modulating the activity of transcription factors such as Tbx1, Gata3 and Foxp3 [22]. In rheumatoid arthritis, decreased EZH2 expression in circulating CD4+ T cells has been linked to impaired Treg differentiation [23]. Similarly, in multiple sclerosis, reduced EZH2 expression in circulating CD4+ and CD8+ T cells of untreated patients has been reported, further emphasising the importance of EZH2 in immune regulation [24]. Conversely, EZH2 expression is increased in CD4+ T cells and CD14+ monocytes in sepsis, suggesting a disease‐specific role for EZH2 in immune‐mediated conditions [25]. These findings highlight the multifaceted and context‐dependent role of EZH2 in modulating immune responses across different diseases.

Despite the well‐established roles of T cells and monocyte‐derived cells in psoriasis, the role of EZH2 in these immune subsets remains unknown. Considering the significance of EZH2 in other autoimmune and inflammatory diseases, investigating its expression in immune subsets of psoriasis patients may uncover commonalities and differences with other immune‐mediated diseases. This study aimed to elucidate the expression patterns of EZH2 in peripheral blood mononuclear cells (PBMCs) from psoriasis patients, with a focus on CD4+ T cells, CD8+ T cells and monocytes. Furthermore, we evaluated whether EZH2 expression correlates with disease severity to assess its potential as a biomarker in psoriasis.

## 2 Materials and Methods

### 2.1 Patients and Healthy Controls

Samples were collected from 40 patients with psoriasis and 18 healthy counterparts matched for sex and age after written informed consent was obtained (Table 1). None of the patients had received treatment with biological agents within 6 months before sample collection, except for topical ointments. Almost all cases used topical steroids or combination products of steroids and active vitamin D3 analogs at the time of blood sample collection. The most frequently used topical agents included calcipotriol/betamethasone dipropionate and clobetasol propionate. Baseline information included age, gender and duration of disease. Disease severity was assessed by dermatologists using the Psoriasis Area Severity Index (PASI) score. There was no apparent correlation between high PASI scores and the frequency of topical medication use. The University of Tokyo Medical Ethics Committee approved all studies described (No. 0695 and 2022272NI), and this study was conducted in accordance with the principles of the Declaration of Helsinki.

**TABLE 1:** Baseline clinical characteristics of psoriasis patients and healthy counterparts.

	Pso	HC	*p*
Number of patients	40	18	—
Sex, M/F, No.	30/10	14/4	> 0.99
Age, years (median, IQR)	52.5 (40.25–60.75)	55.5 (30.75–70)	0.75
PASI (median, IQR)	12.7 (7.4–21.3)		
Disease duration, years (median, IQR)	8.0 (4.0–20.0)		

Abbreviations: HC, healthy counterparts; IQR, interquartile range; PASI, Psoriasis Area Severity Index score; Pso, psoriasis.

### 2.2 Isolation of Peripheral Blood Mononuclear Cells

Primary blood mononuclear cells (PBMCs), including lymphocytes and monocytes, were isolated from psoriasis patients and healthy donors. Blood samples were diluted in 2% FBS/PBS and centrifuged at 800 x g for 15 min at room temperature using Lymphoprep Tube (Serumwerk Bernburg AG, Bernburg, Germany), according to the manufacturer's instructions. The pelleted cells of mononuclear cell fractions were stored at −80°C until further analysis.

### 2.3 Flow Cytometric Analysis

PBMCs were thawed and stained for cell surface markers using fluorescent‐labelled monoclonal antibodies. For lymphocyte subsets, we used labelled BV510 anti‐CD3 antibody (clone OKT3), labelled PECy5 anti‐CD4 antibody (clone RPA‐T4), labelled PE/Dazzle 594 anti‐CD8 antibody (clone one SK1), labelled APCCy7 anti‐CD25 antibody (clone bc96) and labelled Alexa Flour700 anti‐CD45RA antibody (clone HI100); for monocyte subsets, labelled FITC anti‐CD14 antibody (clone HCD14) and labelled APC anti‐CD16 antibody (clone eBioCB16). All antibodies were purchased from BioLegend (San Diego, CA) and eBioscience (San Diego, CA). Subsequently, cells were fixed and permeabilized, followed by intracellular staining for labelled PE anti‐EZH2 antibody (clone M‐T271, BD Bioscience, San Jose, CA) using the FOXP3/Transcription Factor Staining Buffer Set (Thermo Fisher Scientific, Waltham, MA), according to the manufacturer's protocol. The stained cells were analysed using a Cyto FLEX S System (Beckman Coulter, Brea, CA), and the data were processed using FlowJo X software (FlowJo, Ashland, OR). The mean fluorescence intensity (MFI) was used as an indicator of EZH2 expression.

### 2.4 Functional Assays in Primary Human CD8 ^+^ T Cells

PBMCs were cultured for 72 h in RPMI‐1640 medium containing 10% FBS and *Dynabeads Human T‐Activator CD3/CD28* (Thermo Fisher Scientific, Waltham, MA). Cells were treated with the selective EZH2 inhibitor tazemetostat (5 μM; Selleck Chemicals, Houston, TX) or vehicle for 1 h in the presence or absence of recombinant human IL‐1β (10 ng/mL) and IL‐23 (10 ng/mL) (PeproTech, Rocky Hill, NJ). After 72 h, T cells were harvested, and IL17A expression was analysed by qPCR. Primer sequences were as follows: IL17A forward 5′‐AGATTACTACAACCGATCCACCT‐3′, reverse 5′‐GGGGACAGAGTTCATGTGGTA‐3′; GAPDH forward 5′‐CTGGGCTACACTGAGCACC‐3′, reverse 5′‐AAGTGGTCGTTGAGGGCAATG‐3′.

### 2.5 Immunofluorescence Staining

Formalin‐fixed paraffin‐embedded skin sections were deparaffinised and permeabilized with 0.2% Triton‐X 100 in PBS. After heat‐induced antigen retrieval with a citrate‐based antigen unmasking solution (Vector Laboratories, Burlingame, CA), sections were blocked with 5% BSA and 5% normal goat serum in PBS for 1 h at room temperature. The sections were then incubated with primary antibodies against EZH2 (abcam, Cambridge, UK) and CD8 (Proteintech, Rosemont, IL) or CD14 (Cell Signalling Technology, Danvers, MA) at 4°C overnight, followed by 2 h of incubation at room temperature with matched secondary antibodies (Thermo Fisher Scientific, Waltham, MA). Immunofluorescent images were obtained using an all‐in‐one fluorescence microscope BZ‐X810 (Keyence, Itasca, IL).

### 2.6 Statistical Analysis

Statistical analyses were performed using GraphPad Prism version 9 (GraphPad Software Inc., San Diego, CA). Mann–Whitney *U*‐test was conducted for a two‐group comparison. Spearman's rank correlation coefficient was used for the evaluation of correlations between two continuous variables. Two‐way ANOVA followed by Sidak's multiple comparisons test was used for factorial experiments with two variables. Multiple regression analysis was performed to eliminate potential confounding effects of sex, age and disease duration. The analysis was performed using the EZR software program [26].

### 2.7 Use of Artificial Intelligence (AI) Tools

DeepL and ChatGPT were used exclusively for English language editing and proofreading. The authors reviewed, edited and approved all edits and suggestions provided by these AI tools.

## 3 Results

### 3.1 Peripheral Blood Immune Cell Profiling in Psoriasis and Its Correlation With Disease Severity

**FIGURE 1:**
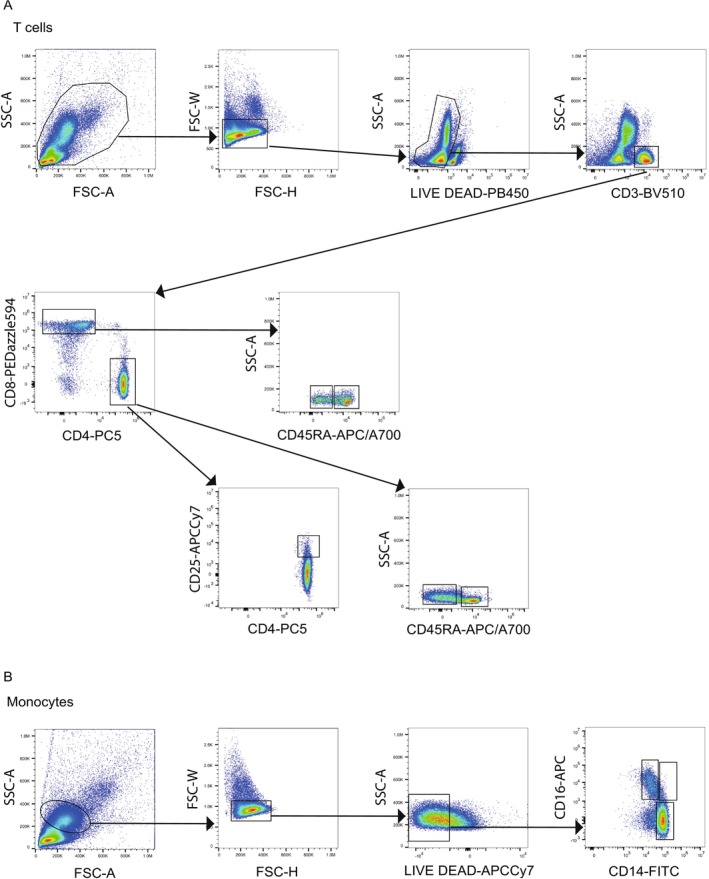
Gating strategy for immune cell subsets. Representative flow cytometry plots illustrating gating strategies for T cells and monocytes. Peripheral blood mononuclear cells (PBMCs) were thawed and stained with cell surface antibodies, followed by intracellular staining with an EZH2 antibody, as described in the Methods section. For T cells (A): Doublet removal was performed using forward scatter‐height (FSC‐H) versus FSC‐A gating. Viable cells were then identified by LIVE/DEAD staining, followed by selection of CD3‐positive cells and separation of CD4‐positive and CD8‐positive fractions. Among CD4‐positive cells, CD25‐positive cells were identified as regulatory T cells, CD45RA‐positive cells as naive CD4, and CD45RA‐negative cells as memory CD4. Among CD8‐positive cells, CD45RA‐positive cells were identified as naive CD8, and CD45RA‐negative cells as memory CD8. For monocytes (B): Cells were similarly gated using FSC‐A and SSC‐A to select monocytes, followed by doublet exclusion (FSC‐H versus FSC‐A). Viable monocytes were determined by LIVE/DEAD staining. The monocyte subpopulations were further identified based on the expression of CD14 and CD16 as follows: CD14‐positive, CD16‐negative cells were classified as classical monocytes, CD14‐positive, CD16‐positive cells as intermediate monocytes and CD14‐negative, CD16‐positive cells as non‐classical monocytes.

In this study, we first analysed the blood profiles of patients with psoriasis. Blood samples were collected from untreated psoriasis patients and from matched healthy controls. Peripheral blood immune cells were analysed by flow cytometry, and a detailed gating strategy was employed to identify specific immune cell subsets (Figure 1A,B). The analysis focused on the proportion of CD4+ naïve and memory T cells, CD8+ naïve and memory T cells and regulatory T cells (Tregs; CD4+CD25+). Additionally, monocytes were classified into three subsets based on the expression of CD14 and CD16: classical monocytes (CD14+ CD16−), non‐classical monocytes (CD14− CD16+) and intermediate monocytes (CD14+ CD16+).

**FIGURE 2:**
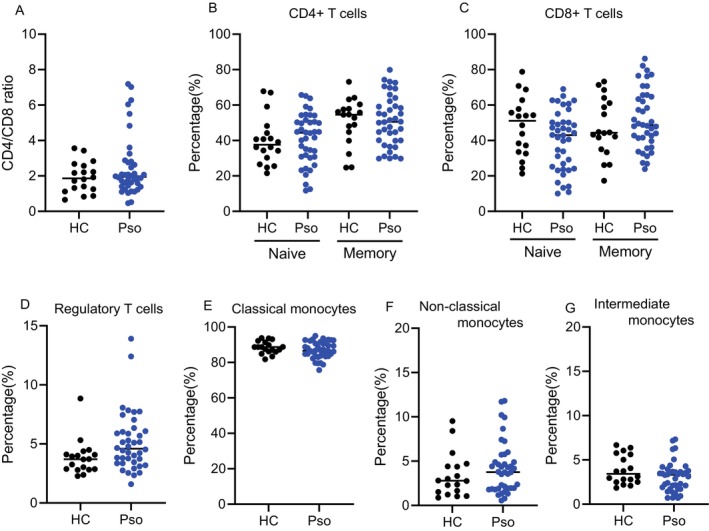
Immune profiles of peripheral blood mononuclear cells from healthy controls and patients with psoriasis. Immune cell subset profiles, including CD4/CD8 ratios, T cell subsets and monocyte subsets, were compared between healthy individuals (HC, *N* = 18) and patients with psoriasis (Pso, *N* = 40). (A) CD4/CD8 ratio. Proportions of (B) naïve CD4+ T cells (CD4+CD45RA+) and memory CD4+ T cells (CD4+CD45RA−), (C) naïve CD8+ T cells (CD8+CD45RA+) and memory CD8+ T cells (CD8+CD45RA−), (D) regulatory T cells (CD4+CD25+), (E) classical monocytes (CD14+CD16−), (F) non‐classical monocytes (CD14−CD16+) and (G) intermediate monocytes (CD14+CD16+). The horizontal lines represent the mean values. Comparisons were assessed by the Mann–Whitney test; no significant differences were observed unless otherwise noted.

The proportions of these immune cell subsets were compared between psoriasis patients and healthy controls (Figure 2A–G), where no significant differences were observed in any subset. Furthermore, no significant correlations between PASI score and the proportions of any immune cell subset were observed.

### 3.2 EZH2 Expression and Its Functional Roles in CD8‐Positive T Cell Subsets

**FIGURE 3:**
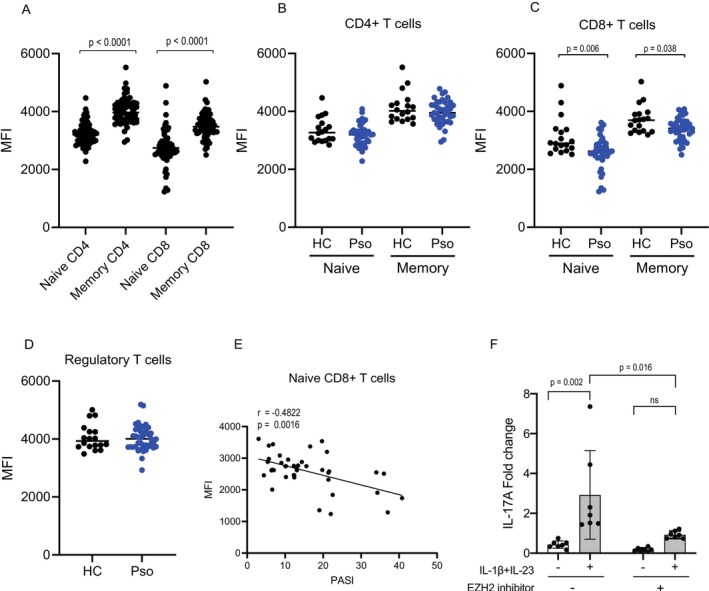
Decreased EZH2 expression in CD8 T cells in patients with psoriasis. EZH2 expression, measured as mean fluorescence intensity (MFI), was evaluated in peripheral T cell subsets from healthy individuals (HC, *N* = 18) and patients with psoriasis (Pso, *N* = 40). (A) MFI of EZH2 in memory and naïve CD4+ and CD8+ cells, combined data from both HC and Pso groups. Comparisons of EZH2 MFI were shown in (B) naïve CD4+ T cells (CD4+CD45RA+) and memory CD4+ T cells (CD4+CD45RA−), (C) naïve CD8+ T cells (CD8+CD45RA+) and memory CD8+ T cells (CD8+CD45RA−), (D) regulatory T cells (CD4+CD25+). Correlation between Psoriasis Area and Severity Index (PASI) scores and EZH2 MFI were shown in (E) naïve CD8 T cells. The horizontal lines represent the mean values. Comparisons were assessed by Mann–Whitney test; no significant differences were observed unless otherwise noted. Correlations were evaluated by Spearman's rank test. (F) PBMCs were stimulated with anti‐CD3/CD28 in the presence or absence of IL‐23 (10 ng/mL) and IL‐1β (10 ng/mL), with or without pre‐treatment of the selective EZH2 inhibitor tazemetostat. IL‐17A mRNA expression was quantified by qPCR and presented as relative expression normalised to GAPDH. Data are presented as mean ± SEM. Statistical significance was determined by two‐way ANOVA followed by Sidak's multiple comparisons test.

Next, we evaluated the expression levels of EZH2 in T cell subsets using the median fluorescence intensity (MFI) as an indicator, an established and validated method in peripheral blood immune cells [23]. Negative control antibodies were included to ensure data reliability (Figure S1A). Analysis of all samples, including both psoriasis patients and healthy controls, revealed significantly higher EZH2 expression in memory CD4+ or CD8+ T cells compared to their naïve counterparts (Figure 3A), suggesting a potential role of EZH2 in T cell differentiation, independent of disease status. Further analysis revealed no significant differences in EZH2 expression levels in CD4+ T cells, including naïve and memory subsets, between psoriasis patients and healthy controls (Figure 3B). Notably, a significant reduction in EZH2 expression, specifically in CD8+ naïve and memory T cells of psoriasis patients, was observed compared to healthy controls (Figure 3C). Furthermore, EZH2 expression levels in regulatory T cells (CD4+CD25+) did not differ significantly between psoriasis patients and healthy controls (Figure 3D). To investigate the potential association between EZH2 expression and disease activity, we examined its correlation with the PASI score. A significant inverse correlation was observed between EZH2 MFI values in CD8+ naïve T cells and the Psoriasis Area and Severity Index (PASI) score (Figure 3E). Additional analyses colour‐coded by sex, age and disease duration are presented in Figure S1E–G. To eliminate potential confounding effects from sex, age and disease duration, we further performed multiple regression analysis. The analysis demonstrated that PASI score remained independently correlated with EZH2 expression (95% CI: −47.09 to −9.99, *p* = 0.003), whereas sex, age and disease duration showed no significant correlation (Table 2). These findings suggest that reduced EZH2 expression in CD8+ naïve T cells may contribute to disease severity, possibly affecting T cell differentiation and function.

**TABLE 2:** Multiple regression analysis by gender, age, disease duration and PASI score.

Predictors	Multivariate		
	Coefficient	95% CI	*p* value
Male sex	87.37	−311.58, 486.31	0.40
Age (years)	4.79	−6.60, 16.17	0.66
Disease duration (years)	−11.34	−26.51, 3.83	0.14
PASI	−28.54	−47.09, −9.99	0.003

Abbreviations: CI, confidence interval; PASI, Psoriasis Area Severity Index score.

To further explore the functional role of EZH2 in CD8^+^ T cells, we next performed a pharmacological inhibition assay using PBMC cultures. Stimulation with anti‐CD3/CD28 in the presence of IL‐23 and IL‐1β robustly induced IL‐17A production. Pre‐treatment with a selective EZH2 inhibitor (tazemetostat) partially but consistently suppressed IL‐17A expression compared with vehicle‐treated controls (Figure 3F). These results indicate that EZH2 activity contributes to the IL‐23/IL‐1β–driven type 3 response.

### 3.3 EZH2 Expression Is Decreased in Monocyte Subsets in Psoriasis

**FIGURE 4:**
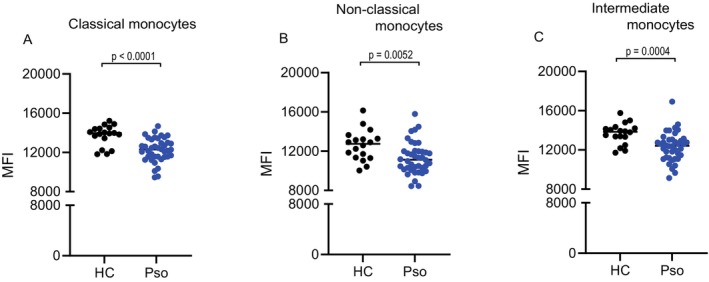
Decreased EZH2 expression in monocytes from patients with psoriasis. EZH2 expression, measured as mean fluorescence intensity (MFI), was evaluated in monocyte subsets from healthy individuals (HC, *N* = 18) and patients with psoriasis (Pso, *N* = 40). Comparisons of EZH2 MFI were shown in (A) classical monocytes (CD14+CD16‐), (B) non‐classical monocytes (CD14−CD16+) and (C) intermediate monocytes (CD14+CD16+). The horizontal lines represent the mean values. Comparisons were assessed by the Mann–Whitney test.

We next assessed the expression levels of EZH2 in monocyte subsets. Monocytes were classified into classical (CD14+ CD16−), non‐classical (CD14− CD16+) and intermediate (CD14+ CD16+) subsets for analysis. Negative control antibodies were included to ensure data reliability (Figure S1B–D). Across all monocyte subsets, EZH2 expression levels were significantly lower in psoriasis patients than in healthy controls (Figure 4A–C). EZH2 expression levels did not significantly correlate with PASI scores in any monocyte subset (classical monocytes: *p* = 0.73; *r* = 0.05, non‐classical monocytes: *p* = 0.90; *r* = 0.02, intermediate monocytes: *p* = 0.96; *r* = 0.22).

### 3.4 EZH2 Positive CD8+ and CD14+ Cells Infiltrate Psoriatic Skin

**FIGURE 5:**
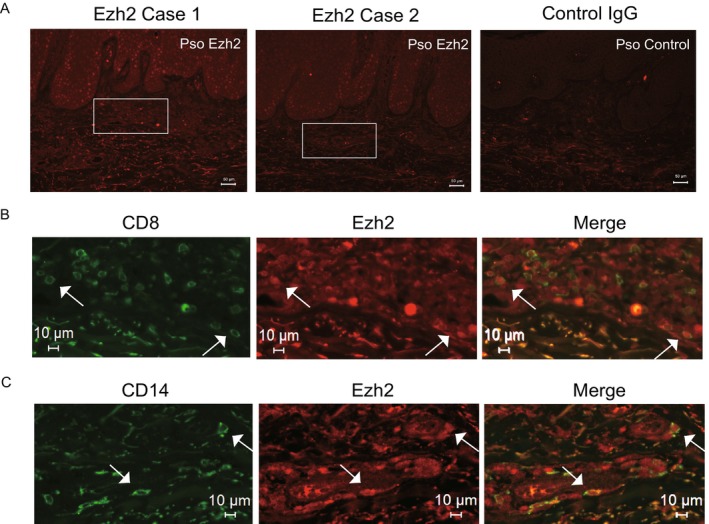
Colocalization of EZH2 with CD8 or CD14 in infiltrating cells from psoriatic skin. (A) Representative low magnification images of EZH2 staining (red) from two independent cases (Case 1 and Case 2) and control IgG staining in psoriatic skin. White boxes indicate areas magnified in panels B and C. Scale bar, 50 μm. (B) High magnification images showing colocalization of EZH2 (red) with infiltrating CD8‐positive T cells (green) in dermal infiltrates. Arrows indicate examples of colocalization. Scale bar, 10 μm. (C) High magnification images showing colocalization of EZH2 (red) with infiltrating CD14‐positive cells (green). Arrows indicate examples of colocalization.

Immunofluorescent staining was then performed to examine the expression and distribution of EZH2 in the psoriatic skin. Representative images of EZH2 staining from two independent cases are shown in Figure 5A, highlighting EZH2 expression (red) in the dermal infiltrates as well as staining also observed in the epidermis. Higher magnification images further revealed that EZH2 expression colocalized with a subset of infiltrating CD8‐positive T cells (green) within dermal regions (Figure 5B). Although CD14‐positive cell infiltration (green) was comparatively sparse relative to CD8‐positive cells, EZH2 expression was similarly observed in a subset of these infiltrating CD14‐positive cells (Figure 5C). These findings indicate a potential role in the local immune response contributing to psoriasis pathogenesis.

## 4 Discussion

This study aimed to evaluate the expression of EZH2 across immune cell subsets in the peripheral blood of patients with psoriasis. While no significant differences were observed in the overall immune profiles between patients with psoriasis and healthy controls, EZH2 expression was notably reduced in specific subsets of CD8 T cells and monocytes in psoriasis patients. In particular, a significant inverse correlation was found between EZH2 expression in CD8 T cells and PASI scores, suggesting a potential link between disease severity and epigenetic regulation in these cells.

The function of EZH2 in T cells has been extensively studied in CD4 T cells; however, its importance in CD8 T cells has recently gained increasing attention [27]. EZH2 has been reported to suppress the expression of cyclin‐dependent kinase inhibitors, such as Cdkn2a and Cdkn1c, thereby promoting the proliferation and survival of CD8 T cells [28]. Additionally, EZH2 influences the differentiation of CD8+ T cells into effector and memory cells [29, 30]. Our findings align with these previous reports, as we observed that EZH2 expression was higher in memory CD8 T cells compared to naïve CD8 T cells. Furthermore, psoriasis patients exhibited a reduction in EZH2 expression in both naïve and memory CD8 T cells, suggesting that epigenetic dysregulation may occur early in T cell differentiation. Comparable findings have been reported in rheumatoid arthritis, where EZH2 downregulation has been reported in naïve CD4 T cells [23], indicating that epigenetic abnormalities in immune cells may be an early event in chronic inflammatory diseases. This study also observed a statistically significant inverse correlation between EZH2 expression in naïve CD8 T cells and PASI scores and a similar trend in memory CD8 T cells. These findings suggest that more severe psoriasis is associated with lower EZH2 expression in CD8 T cells, potentially affecting immunological conditions by shaping the epigenetic landscape of CD8+ T cells.

While EZH2 function in monocytes remains relatively unexplored, prior studies investigating EZH2 expression in PBMCs from RA patients have reported a trend toward reduced EZH2 expression [23], although statistical significance was not reached. EZH2 is known to function as an epigenetic regulator in monocyte differentiation and macrophage polarisation [31]. It has been implicated as an epigenetic checkpoint in monocyte differentiation, facilitating monocyte repair functions and playing a protective role in preventing infarct expansion and cardiac dysfunction post‐myocardial infarction. In acute inflammatory conditions such as sepsis, EZH2 expression in monocytes is elevated [25], which may indicate enhancing repair functions to mitigate organ damage. This study observed decreased EZH2 expression in monocytes from psoriasis patients, suggesting a potential dysregulation in monocyte‐to‐macrophage differentiation and polarisation. This dysregulation could lead to an imbalance in M1/M2 macrophage polarisation, favouring an increase in pro‐inflammatory M1 macrophages, thereby contributing to the persistence of chronic inflammation in psoriasis. However, the precise role of EZH2 in monocyte‐mediated inflammation in psoriasis warrants further investigation.

Our findings regarding reduced EZH2 expression in peripheral immune subsets contrast with previous studies demonstrating increased EZH2 expression in psoriatic epidermis. Specifically, Quah et al. [32] and others have reported elevated EZH2 levels in lesional skin, highlighting potential tissue‐specific differences in EZH2 regulation. The apparent discrepancy between reduced EZH2 expression in circulating immune cells and increased expression in psoriatic lesions may reflect compartment‐ and context‐dependent regulation. Local factors within the lesional microenvironment, such as pro‐inflammatory cytokines (IL‐17, IL‐23, TNF‐α) and keratinocyte‐derived signals, may sustain or enhance EZH2 expression in skin‐resident immune cells, whereas circulating CD8^+^ T cells and monocytes are exposed to distinct systemic cues that may lead to reduced expression. Although the precise mechanisms underlying this tissue‐specific regulation remain unclear and represent a limitation of our study, the divergence suggests that EZH2 is dynamically regulated according to cellular niche and inflammatory context. It also remains a possibility that reduced EZH2 expression in circulating CD8^+^ T cells reflects a compensatory or secondary adaptation to chronic systemic inflammation rather than a primary pathogenic driver. Future studies will be required to clarify whether EZH2 downregulation is causative in psoriasis pathogenesis or represents an adaptive response to the inflammatory milieu.

Additionally, Zhang et al. [33] observed no significant change in H3K27me3 levels in PBMCs from psoriasis patients, which seemingly contrasts our results. Given that H3K27me3 is dynamically modulated by multiple enzymes, including EZH1, EZH2 and demethylases such as UTX and JMJD3, a direct relationship between EZH2 expression alone and overall H3K27me3 status might not always be evident. Future comprehensive analyses incorporating other epigenetic modifiers and comparing tissue‐specific versus circulating immune cell populations will be critical to fully understand EZH2's role and its complex epigenetic regulation in psoriasis pathogenesis.

In conclusion, our findings suggest that EZH2 downregulation may play a role in psoriasis pathogenesis, particularly through its effects on the function and differentiation of CD8 T cells and monocytes. Future studies should explore the mechanistic links between EZH2 expression, immune cell dysfunction and chronic inflammation to better understand its potential as a therapeutic target in psoriasis.

## Author Contributions

Toyoki Yamamoto and Sayaka Shibata designed the experiments. Toyoki Yamamoto and Sayaka Shibata wrote the manuscript. Toyoki Yamamoto performed most of the experiments with contributions from Lixin Li, Asumi Koyama and Rino Toyoshima. Toyoki Yamamoto and Sayaka Shibata analysed the data. Shinichi Sato supervised the project. All authors discussed the results and commented on the manuscript.

## Conflicts of Interest

The authors declare no conflicts of interest.

## Supporting information


**Figure S1:** Correlation analyses and representative histograms of EZH2 expression in CD8+ T cells and monocyte subsets.

## Data Availability

The data that support the findings of this study are available on request from the corresponding author. The data are not publicly available due to privacy or ethical restrictions.
