# Supplementary material for: Reduced EZH2 Expression in Circulating CD8‐Positive T Cells and Monocytes in Psoriasis
Source: Exp Dermatol. 2026 Jan 10;35(1):e70207. doi: 10.1111/exd.70207 (PMC12790368; doi:10.1111/exd.70207)
Supplement: Supplementary file 1 — Figure S1: Correlation analyses and representative histograms of EZH2 expression in CD8+ T cells and monocyte subsets. [file EXD-35-e70207-s001.docx]

**
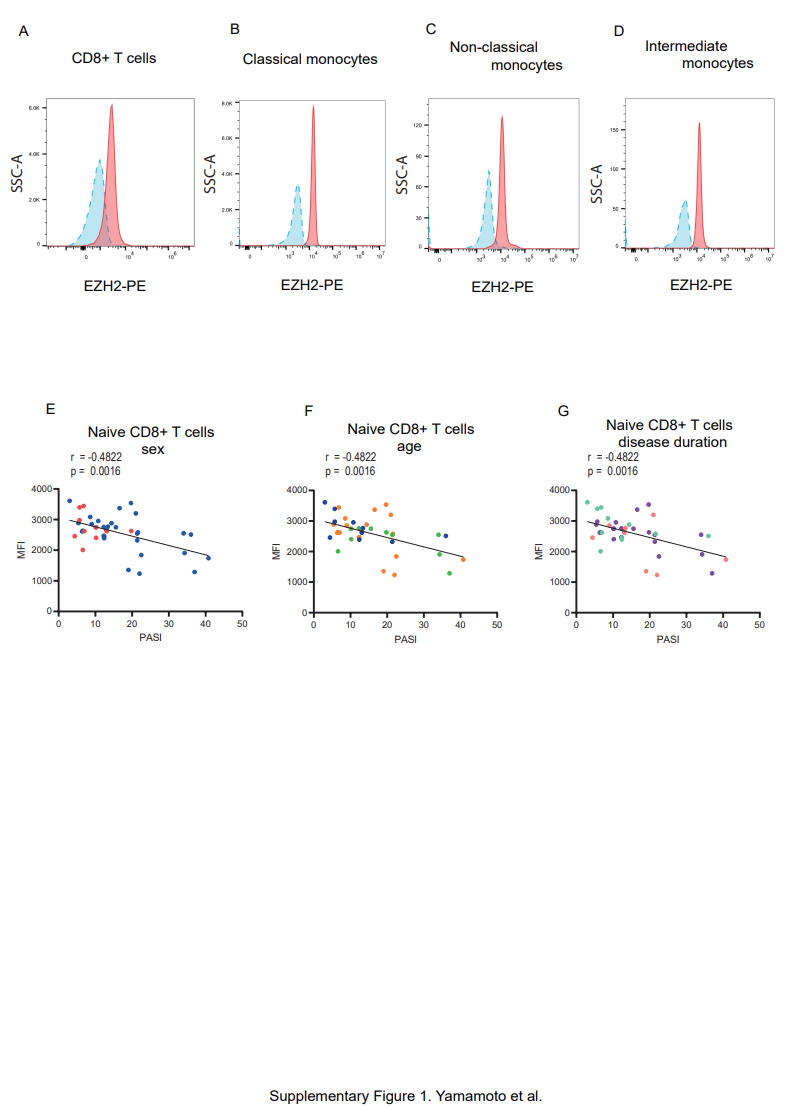
**

**Supplementary Figure 1. Correlation analyses and representative histograms of EZH2 expression in CD8+ T cells and monocyte subsets**

(A–D) Representative histograms showing EZH2 expression (red-filled) compared to corresponding control IgG staining (blue dashed line) in (A) CD8+ T cells, (B) classical monocytes, (C) non-classical monocytes, and (D) intermediate monocytes.

(E–G) Correlation between EZH2 mean fluorescence intensity (MFI) in naïve CD8+ T cells and PASI scores, color-coded by clinical characteristics: (A) sex (male, red; female, blue), (B) age (21–40 years, green; 41–60 years, orange; ≥61 years, blue), and (C) disease duration (<5 years, green; 5–20 years, purple; ≥21 years, red).
